# Best evidence summary on anticoagulant management in patients with cancer-associated venous thromboembolism

**DOI:** 10.1016/j.apjon.2025.100789

**Published:** 2025-09-20

**Authors:** Xinyue Xiang, Yudi Yu, Xiaomei Fang, Tian Zheng, Wenbo Qiao

**Affiliations:** a Department of Nursing, The First Affiliated Hospital, Zhejiang University School of Medicine, Hangzhou, China; b Department of Respiratory medicine, The First Affiliated Hospital, Zhejiang University School of Medicine, Hangzhou, China

**Keywords:** Cancer, Venous thromboembolism, Anticoagulation, Evidence-based nursing

## Abstract

**Objective:**

To retrieve, evaluate, and summarize the best evidence for anticoagulant management in patients with cancer-associated venous thromboembolism (VTE).

**Methods:**

This was an evidence summary study conducted in strict accordance with the reporting standards of the Fudan University Center for Evidence-Based Nursing. According to the evidence pyramid “6S” model, the search was performed from top to bottom. The following databases were searched: BMJ Best Practice, UpToDate, Joanna Briggs Institute, Cochrane Library, Guidelines International Network, National Institute for Health and Clinical Excellence, Scottish Intercollegiate Guidelines Network, Registered Nurses Association of Ontario, YiMaiTong Guidelines Network, PubMed, Embase, Web of Science, Sinomed, China National Knowledge Infrastructure (CNKI), Wanfang, and VIP. Professional association websites included those of the American Society of Clinical Oncology, American Society of Hematology, British Society for Haematology, and the Scientific and Standardization Committee of the International Society on Thrombosis and Haemostasis. The search period was from the inception of each database to October 2024. Literature types included clinical practice guidelines, clinical decisions, expert consensuses, systematic reviews, and randomized controlled trials (RCTs).

**Results:**

A total of 19 studies were included, comprising three clinical decisions, 10 guidelines, one expert consensus, four systematic reviews, and one RCT. Thirty-four pieces of evidence were synthesized across eight aspects: risk factor assessment, timing of anticoagulation, drug selection, treatment duration, management of recurrence, safety monitoring, health education, and follow-up care.

**Conclusions:**

This study summarizes the best evidence for anticoagulant management in patients with cancer-associated VTE. When applying this evidence clinically, health care providers should thoroughly evaluate the feasibility and appropriateness of each recommendation to develop clinical decisions tailored to China's national conditions and health care resources.

**Systematic review registration:**

This study has been registered on the Fudan University Centre for Evidence-based Nursing (Registration No. ES20244015).

## Introduction

Venous thromboembolism (VTE), a common complication among cancer patients, encompasses deep vein thrombosis (DVT) and pulmonary embolism (PE), and ranks as the second leading cause of mortality following disease progression.^1^^,^^2^ Due to the hypercoagulable state, endothelial damage, and venous stasis caused by the disease itself and treatment (such as surgery, chemotherapy, and the use of targeted drugs) in cancer patients, their risk of VTE is 9 times higher than that of the general population.^3^^,^^4^ Studies have shown that the incidence of VTE in China has increased from 3.2 per 100,000 to 17.5 per 100,000 in the past 10 years.^5^ Specific types of cancer patients, such as those with pancreatic cancer and lung cancer, have even higher risks (the prevalence of pancreatic cancer is 8.5%, and 9.7% of lung cancer patients develop VTE).^6^^,^^7^ VTE not only elevates hospital readmission rates, recurrence of thrombotic events, and the likelihood of major bleeding episodes but also exacerbates health care burdens and significantly compromises patients’ quality of life.^8^ Therefore, effective preventive and therapeutic interventions for cancer-associated VTE hold substantial clinical importance.

Currently, the guidelines clearly recommend anticoagulants as the core intervention for the prevention and treatment of cancer-associated thrombosis (CAT).^9^ However, there is a significant gap between clinical reality and guideline recommendations.^5^^,^^10^^,^^11^ CAT remains insufficiently recognized among cancer patients, and the utilization rate of preventive measures is far from ideal.^12^^,^^13^ Meanwhile, there are substantial variations in the management approaches of CAT in real-world clinical practice, which not only reflects the complexity of individualized care but also exposes the dilemma of clinical decision-making lacking unified and reliable guidance.^14^ Although anticoagulant therapy serves as the cornerstone for the management of CAT, the unique characteristics of cancer patients pose multiple challenges to treatment. Studies have shown that even with standardized anticoagulation, this group still faces a higher risk of thrombus recurrence and bleeding.^15^ In fact, individual differences in tumor type, disease stage, underlying comorbidities, antineoplastic treatment regimens, and prognosis may all influence decisions regarding the implementation of thrombosis prevention, further exacerbating the complexity and uncertainty of treatment.^16^^,^^17^

The number of domestic and international literature on anticoagulation management in cancer patients has been increasing. However, the quality of evidence from these literature varies considerably, the content is relatively scattered, and the implementation effect of the findings is suboptimal.^18^ Existing studies mostly focus on comparing the efficacy, safety, and economic benefits of different types of anticoagulants. Nevertheless, they lack precise guidance tailored to the individual characteristics of cancer patients, such as tumor type, comorbidities, and drug interactions. This makes it difficult to meet the needs of individualized treatment in clinical practice.^19^, ^20^, ^21^, ^22^ Secondly, there is no consensus on the optimal duration of anticoagulant therapy. Most studies only focus on short-term efficacy and lack long-term follow-up data, failing to provide clear recommendations for the duration of continuous treatment in clinical settings.^23^^,^^24^ Further high-quality evidence-based medicine is still needed to determine how to optimize anticoagulation strategies to balance benefits and risks.

Given the high burden of cancer-related VTE, the complexity of anticoagulation management, and the limitations of existing reviews and guidelines, this study aims to systematically search for relevant domestic and foreign literature on the assessment of VTE anticoagulation risks, drug selection, anticoagulation duration, and health education for cancer patients, in order to provide scientific basis for medical staff to implement care interventions.

## Methods

### Establishment of the problem

The evidence-based problem was constructed using the PIPOST model,^25^ with the following components: (1) Population (P): The cancer patients, both with and without VTE; (2) Intervention (I): Management of cancer-related VTE; (3) Profession (P): Physicians, nurses, patients, and family members.; (4) Outcome (O): Adverse reactions associated with anticoagulant medications, risks of recurrence, and instances of bleeding; (5) Setting (S): Home, nursing homes, retirement communities; (6) Type of Evidence (T): Clinical decision-making resources, guidelines, systematic reviews, expert consensus, meta-analysis, randomized controlled trials (RCTs), among others. This study was registered at the Fudan University Center for Evidence-Based Nursing (Registration No. ES20244015).

### Search strategy

According to the 6S evidence resource model, evidence retrieval is conducted from the top down.^26^ Computerized searches were executed across various platforms, including UpToDate, BMJ Best Practice, the Guidelines International Network (GIN), the Scottish Intercollegiate Guidelines Network (SIGN), the Registered Nurses Association of Ontario (RNAO), the National Guideline Clearinghouse (NGC), the National Institute for Health and Care Excellence (NICE) guideline database, and Medlive. The English-language databases utilized included PubMed, Web of Science, the Cochrane Library, and Embase, while the Chinese-language databases included the China National Knowledge Infrastructure (CNKI), Wanfang, VIP, and China Biology Medicine. Additionally, professional association websites such as the International Thrombosis and Hemostasis (ITAC), the American Society of Clinical Oncology (ASCO), the American Society of Hematology (ASH), and the National Comprehensive Cancer Network (NCCN) were also included in the search. The search strategy integrated both free-text terms and subject headings.

The objective is to utilize a combination of Medical Subject Headings (MeSH) and free-text terms to formulate search queries. For instance, in the context of PubMed, the search strategy can be articulated as follows: (((("Neoplasms" [MeSH]) OR ("Tumor" [Ti/Ab]) OR ("Cancer" [Ti/Ab]) OR ("Malignant Neoplasm" [Ti/Ab]) OR ("Malignancy" [Ti/Ab])) AND ((("Venous Thrombosis" [MeSH]) OR ("Pulmonary Embolism" [MeSH])) OR ("Phlebothrombosis" [Ti/Ab]) OR ("Thrombosis, Venous" [Ti/Ab]) OR ("Deep Vein Thrombosis" [Ti/Ab]) OR ("Deep-Venous Thrombosis" [Ti/Ab]) OR ("Venous Thromboses, Deep" [Ti/Ab]) OR ("Pulmonary Thromboembolism" [Ti/Ab]) OR ("Thromboembolism, Pulmonary" [Ti/Ab]))) AND ((("Heparin, Low-Molecular-Weight" [MeSH]) OR ("Factor Xa Inhibitors" [MeSH]) OR ("Warfarin" [MeSH]) OR ("Heparin" [MeSH]) OR ("Fondaparinux" [MeSH]) OR ("Dalteparin" [MeSH])) OR ("LMWH" [Ti/Ab]) OR ("Low Molecular Weight Heparin" [Ti/Ab]) OR ("Anticoagulants, Direct-Acting Oral" [Ti/Ab]) OR ("New Oral Anticoagulant" [Ti/Ab]) OR ("Unfractionated Heparin" [Ti/Ab]) OR ("Apixaban" [Ti/Ab]) OR ("Edoxaban" [Ti/Ab]) OR ("Vitamin K Antagonist" [Ti/Ab]))))). The search strategies for each database are detailed in Appendix 1.

### Study inclusion and exclusion criteria

**The inclusion criteria were as follows:** (1) Participants must be cancer patients, and the research must focus on anticoagulant management for VTE; (2) Eligible study types include clinical decision-making studies, guidelines, systematic reviews, evidence summaries, expert consensus statements, and randomized controlled trials; (3) The languages of the included literature were Chinese and English.

**The exclusion criteria were as follows:** (1) Incomplete literature information or duplicates; (2) Inaccessible full texts; (3) Studies failing quality assessment.

### Literature quality assessment

(1) Clinical decision-making evidence, lacking an internationally recognized assessment tool, was directly classified as high-quality evidence when sourced from authoritative databases, as indicated in the relevant literature. (2) The guidelines were evaluated using the Appraisal of Guidelines for Research and Evaluation (AGREE II),^27^ updated in 2012 in the United Kingdom. This instrument includes six dimensions and 23 items, each rated on a scale from 1 to 7, ranging from “strongly disagree” to “strongly agree”. Higher scores indicate better compliance with the item. (3) Expert consensus, Systematic reviews, and RCTs were evaluated based on the criteria of the authentic assessment tool (2016) from the Australian JBI Center for Evidence-Based Health Care.^28^^,^^29^ (4) For systematic reviews, the AMSTAR 2 tool was utilized for evaluation.^30^

The quality of literature will be independently assessed by two master's-level nursing researchers who have received training in evidence-based nursing. In case of discrepancies in the assessment results, a decision will be made after discussion by the evidence-based nursing team. This team consists of two master's supervisors in nursing, one head nurse, one deputy head nurse, one senior nurse practitioner, and two master's-level nursing researchers. When conclusions from different sources of evidence conflict, the principles of prioritizing evidence-based evidence, high-quality evidence, and the most recently published authoritative literature shall be followed.

### Evidence extraction, translation, integration, and grading

Two researchers will independently screen the retrieved literature in accordance with the established inclusion and exclusion criteria. In case of disagreements during the screening process, they will first strive to reach a consensus through in-depth discussions. If consensus cannot be achieved after discussions, a third researcher will be consulted to resolve the dispute. After completing the literature screening, the aforementioned two researchers will independently extract data using the same standardized data extraction form, with both remaining blinded to each other's extraction results during this process. The extracted data include the basic characteristics of the studies, specifically covering the first author's name and affiliated institution, the year of publication, the source of the literature, the type of evidence, and the research topic.

To ensure the accuracy of evidence translation, English recommendations or research conclusions extracted from various literature should be translated independently by two researchers proficient in foreign languages. After the translation is completed, the two versions shall be compared. In case of inconsistencies, discussions shall be held between the two researchers or a third researcher shall be consulted to resolve the discrepancies. Subsequently, both the original English manuscripts and the translated versions shall be submitted to the steering group for review, and the final translated manuscripts shall be finalized. This multi-step translation and review process minimizes translation bias and ensures the reliability of the summarized evidence.

When summarizing the evidence, the following principles should be followed. For different sources of evidence that are consistent or complementary in content, they should be combined and presented together. For example, recommendations from UpToDate suggest that for patients with stable hemodynamics and cancer-related VTE, if there is no severe renal dysfunction (creatinine clearance rate ≥ 30 mL/min) and no contraindications, it is recommended to directly use DOACs instead of low molecular weight heparin or traditional anticoagulation regimens [that is, intravenous administration of unfractionated heparin followed by warfarin]. Meanwhile, the International Thrombosis and Cancer Initiative Clinical Practice Guidelines suggest that if there is severe renal failure (creatinine clearance rate used, followed by early combination with vitamin K antagonists (which can be started from the first day), or LMWH (with anti-Xa factor levels needing to be monitored and the dose adjusted promptly) to prevent cancer-related VTE. The two sets of recommendations are complementary and should be integrated when presented.

The included evidence was graded and recommended by the JBI Evidence and Recommendation Classification System. Based on the type of research design, the evidence level is classified from levels 1–5. At the same time, according to the FAME structure of JBI, the feasibility, appropriateness, meaningfulness, and effectiveness of the evidence were evaluated, and the recommendation level of the evidence was determined, namely A-level recommendation (strong recommendation) and B-level recommendation (weak recommendation).^31^

## Results

### General characteristics of the included literature

**Fig. 1:**
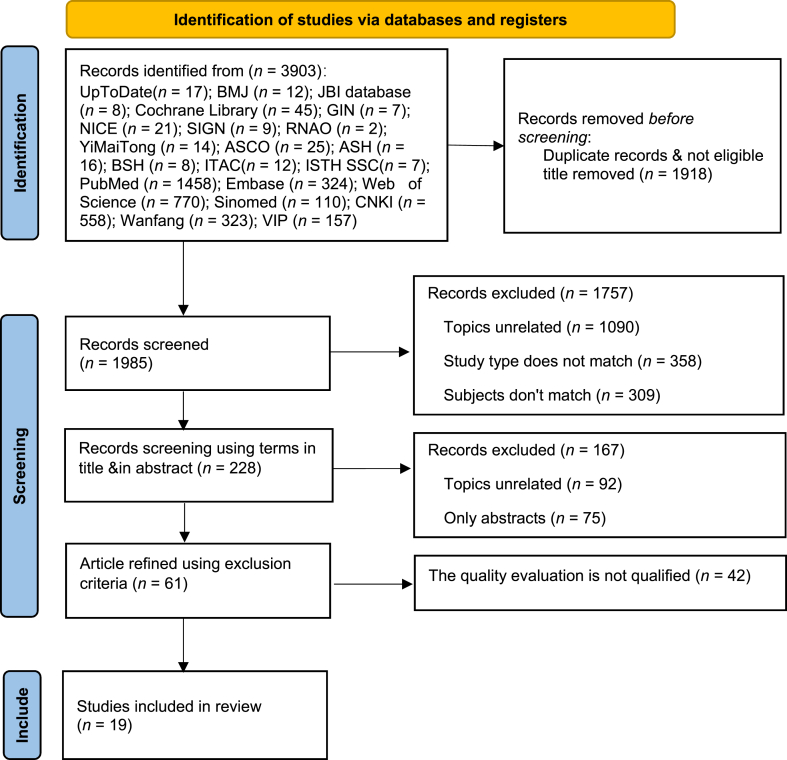
Literature screening flow chart. GIN, Guidelines International Network; NICE, National Institute for Health and Care Excellence; SIGN, Scottish Intercollegiate Guidelines Network; RNAO, Registered Nurses Association of Ontario; ASCO, American Society of Clinical Oncology; ASH, American Society of Hematology; ITAC, International Thrombosis and Hemostasis; CNKI, China National Knowledge Infrastructure.

A preliminary search identified a total of 3903 articles, which was subsequently reduced to 1985 following the removal of duplicates. Through an examination of the titles and abstracts, 1757 articles were excluded, resulting in a final selection of 19 articles for inclusion. This selection comprised 3 clinical decisions,^32^, ^33^, ^34^ 10 guidelines,^35^, ^36^, ^37^, ^38^, ^39^, ^40^, ^41^, ^42^, ^43^, ^44^ 1 expert consensus,^45^ 4 systematic reviews,^46^, ^47^, ^48^, ^49^ and 1 RCT.^50^ The literature screening process is shown in Fig. 1, and the general characteristics of the included literature are shown in Table 1.

**Table 1:** General information included in the studies (*N* ​= ​19).

Included literature	Publish year	Source	Type	Topic
Kenneth^32^	2023	Up to Date	Clinical decisions	Anticoagulant therapy of adult malignant tumors with VTE
Kenneth^33^	2023	Up to Date	Clinical decisions	Risk and prevention of cancer-associated VTE in adults
Thita^34^	2022	Up to Date	Clinical decisions	Causes and mechanisms of cancer-related hypercoagulability
Chinese Society of clinical Oncology and thrombosis expert Committee^35^	2019	CNKI	Guidelines	Guidelines for the prevention and treatment of tumor-associated VTE
Key^36^	2023	ASCO	Guidelines	Treatment and prevention of VTE in cancer patients
Farge^37^	2022	PubMed	Guidelines	Treatment and prevention of VTE in cancer patients, including patients with COVID-19
Falanga^38^	2022	PubMed	Guidelines	VTE in cancer patients: ESMO clinical practice guidelines
Farge^39^	2019	PubMed	Guidelines	International clinical practice guidelines for the treatment and prophylaxis of VTE in patients with cancer
Lyman^40^	2021	ASH	Guidelines	Management of VTE: Prevention and treatment in patients with cancer
Khorana^41^	2007	NCCN	Guidelines	Venous thromboembolic disease: Strategies for improving VTE prophylaxis in hospitalized cancer patients
Streiff^42^	2024	PubMed	Guidelines	NCCN clinical practice guidelines in Oncology
Wang^43^	2019	PubMed	Guidelines	The use of direct oral anticoagulants for primary thromboprophylaxis in ambulatory cancer patients
Alikhan^44^	2024	PubMed	Guidelines	Cancer-associated venous thrombosis in adults (second edition): A British Society for Haematology guideline
Ageno^45^	2013	PubMed	Expert consensus	Selection and evaluation of novel oral anticoagulant therapy patients
Frere^46^	2022	PubMed	Systematic reviews	Comparison of DOAC and LMWH to treat cancer-related VTE
Michalopoulou^47^	2023	PubMed	Systematic reviews	Net clinical benefit of DOAC versus conventional anticoagulant therapy in patients with active cancer VTE
Rutjes^48^	2020	Cochrane Library	Systematic reviews	Primary prevention of VTE in outpatient cancer patients undergoing chemotherapy
Kahale^49^	2021	Cochrane Library	Systematic reviews	Oral anticoagulant therapy in cancer patients with no indication of anticoagulant therapy or prophylaxis
Schrag^50^	2023	PubMed	RCT	A comparison in the prevention of cancer VTE recurrence of DOAC and LMWH

ASCO, American Society of Clinical Oncology; ASH, American Society of Hematology; ESMO, European Society for Medical Oncology; NCCN, National Comprehensive Cancer Network; DOAC, Direct oral anticoagulant; RCT, randomized controlled trial; LMWH, low molecular weight heparin; VTE, Venous thromboembolism; COVID-19, coronavirus disease 2019.

### Evaluation of publication quality

#### Quality evaluation of the guidelines

A total of 10 guidelines^35^, ^36^, ^37^, ^38^, ^39^, ^40^, ^41^, ^42^, ^43^, ^44^ were included in this analysis. The standardized percentages for each dimension, along with their corresponding recommendation levels, are presented in Table 2.

**Table 2:** Quality evaluation results of guidelines (*N* ​= ​10).

Guidelines	Percentage of standardization in each domain of the guide (%)						No. of Felds ≥ 60%	No. of Felds ≥ 30%	Recommendation grade
	Scope and grade purpose	Stakeholder involvement	Rigor of development	Clarity of presentation	Applicability	Editorial independence			
Chinese Society of clinical Oncology and thrombosis expert Committee^35^	83.3	88.9	45.8	83.3	47.3	52.8	4	6	B
Key^36^	100.0	94.4	100.0	98.0	87.5	88.9	6	6	A
Farge^37^	98.1	98.1	100.0	100.0	91.7	97.2	6	6	A
Falanga^38^	100.0	100.0	93.8	96.3	86.1	94.4	6	6	A
Farge^39^	92.1	98.0	95.1	100.0	87.5	91.7	6	6	A
Lyman^40^	94.1	92.2	86.1	85.2	94.4	86.1	6	6	A
Khorana^41^	81.5	76.5	93.8	72.5	95.8	76.4	6	6	A
Streiff^42^	83.3	88.9	88.9	77.8	91.2	75.0	6	6	A
Wang^43^	88.2	87.0	90.3	90.7	97.2	80.6	6	6	A
Alikhan^44^	90.2	100.0	91.7	87.0	83.3	77.8	6	6	A

#### Quality evaluation of the expert consensus

A total of one expert consensus^45^ was included in the analysis. All items received a rating of "Yes," indicating a scientifically robust research design and overall high quality, which justified their inclusion.

#### Quality evaluation of the systematic reviews

Four systematic reviews^46^, ^47^, ^48^, ^49^ were included, and Table 3 shows the quality evaluation results for these systematic reviews. See Table 3 for details of the evaluation results.

**Table 3:** Quality evaluation results of included systematic reviews (*N* ​= ​4).

Systematic reviews	Q1	Q2	Q3	Q4	Q5	Q6	Q7	Q8	Q9	Q10	Q11	Q12	Q13	Q14	Q15	Q16
Frere^46^	Y	Y	Y	Y	Y	Y	Y	Y	Y	Y	Y	Y	Y	Y	Y	Y
Michalopoulou^47^	Y	Y	Y	Y	Y	Y	Y	Y	Y	N	Y	Y	N	Y	Y	Y
Rutjes^48^	Y	Y	Y	Y	Y	Y	Y	Y	Y	Y	Y	Y	Y	Y	Y	Y
Kahale^49^	Y	Y	Y	Y	Y	Y	Y	Y	Y	Y	Y	Y	Y	Y	Y	Y

Y, Yes; N, No.

Q1: Did the research questions and inclusion criteria for the review include the components of PICO? Q2: Did the report of the review contain an explicit statement that the review methods were established prior to the conduct of the review and did the report justify any significant deviations from the protocol? Q3: Did the review authors explain their selection of the study designs for inclusion in the review? Q4: Did the review authors use a comprehensive literature search strategy? Q5: Did the review authors perform study selection in duplicate? Q6: Did the review authors perform data extraction in duplicate? Q7: Did the review authors provide a list of excluded studies and justify the exclusions? Q8: Did the review authors describe the included studies in adequate detail? Q9: Did the review authors use a satisfactory technique for assessing the risk of bias (RoB) in individual studies that were included in the review? Q10: Did the review authors report on the sources of funding for the studies included in the review? Q11: If meta-analysis was performed, did the review authors use appropriate methods for statistical combination of results? Q12: If meta-analysis was performed, did the review authors assess the potential impact of RoB in individual studies on the results of the meta-analysis or other evidence synthesis? Q13: Did the review authors account for RoB in primary studies when interpreting/discussing the results of the review? Q14: Did the review authors provide a satisfactory explanation for, and discussion of, any heterogeneity observed in the results of the review? Q15: If they performed quantitative synthesis did theMreview authors carry out adequate investigation of publication bias (small study bias) and discuss its likely impact on the results of the review? Q16: Did the review authors report any potential sources of conflict of interest, including any funding they received for conducting the review?.

#### Quality evaluation of the randomized controlled trial

A total of one RCT^50^ was included in this analysis, sourced from PubMed. The study exhibited a comprehensive research design and high quality, thereby meeting the criteria for inclusion.

#### Quality assessment of the clinical decisions

A total of three clinical decisions^32^, ^33^, ^34^ were included in this analysis. The methodological quality of the studies corresponding to the included evidence was assessed by reviewing the original literature, all of which demonstrated high quality, thereby justifying their inclusion.

### Summary and description of evidence

**Fig. 2:**
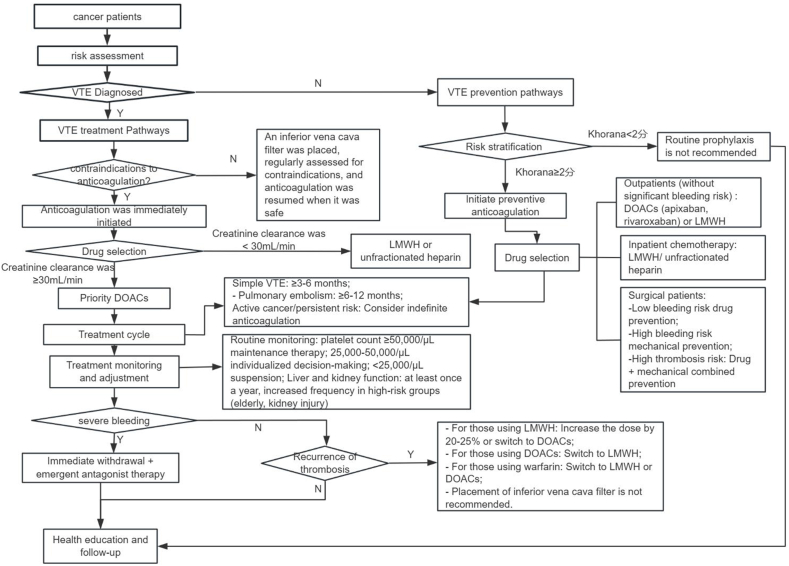
Clinical decision flow chart for Cancer-Associated Anticoagulant Management (based on evidence 5,6,8, 9, 10,13,14,18,21,27,28,30).

In the management of cancer patients with VTE, critical considerations encompass assessing risk, determining the appropriate timing for treatment initiation, selecting suitable pharmacological agents, and ensuring effective safety monitoring. These aspects are initially categorized according to established guidelines or consensus frameworks. A multidisciplinary team, including clinicians, methodological experts, nurses, and patient representatives, is convened to engage in a brainstorming session focused on the research objectives. This collaborative effort aims to identify potential key dimensions, which are subsequently refined through multiple rounds of discussion to ensure their comprehensiveness, independence, and practicality. Eventually, a total of 34 pieces of evidence were summarized from eight aspects: risk factor assessment, anticoagulation timing, drug selection, treatment duration, recurrence treatment, safety monitoring, health education, and follow-up. Please refer to Table 4 and Fig. 2 for details.

**Table 4:** Best evidence on anticoagulant management in patients with cancer-associated VTE.

Evidence aspect	Contents of evidence	Evidence level	Recommended level
**Risk assessment**	1. Risk factors that contribute to hypercoagulable states in patients encompass several categories: Cancer-related factors, which include the type, location, stage, grade, and timing of diagnosis; general individual VTE risk factors: Advanced age, obesity, comorbidities, hereditary thrombophilia, a history of VTE, and COVID-19; and treatment-related factors: Systemic anticancer therapy, including radiotherapy, chemotherapy (e.g., cisplatin), erythropoiesis-stimulating agents, surgical interventions, hospitalization, indwelling catheters, and infusion ports.^33^^,^^34^^,^^39^^,^^42^	1a	A
	2. The assessment content includes the following components: Medical history, physical examination, complete blood count, platelet count, PT, APTT, and serum creatinine levels.^34^^,^^35^	1b	A
	3. Emerging biomarkers: Blood-count parameters: Platelets and leucocytes; D-dimer, plasma troponin, BNP, and NT-proBNP.^35^^,^^39^	1a	A
	4. Imaging techniques include Doppler ultrasonography, CT venography, magnetic resonance imaging (MRI), and traditional venography.^35^		
	5. Risk assessment models: The Khorana risk assessment prediction model is recommended for risk stratification of patients with tumors. However, its ability to accurately discriminate VTE risk in patients with most types of cancer is poor, both in the inpatient setting and in the outpatient setting.^34^^,^^36^^,^^39^^,^^48^	1a	B
**Timing of anticoagulation**	6. For cancer patients who do not have contraindications to anticoagulation, it is recommended that anticoagulant therapy be initiated promptly upon the diagnosis of VTE.^35^	2a	B
	7. Thrombolysis should be evaluated based on the individual circumstances of the patient, with particular consideration given to the associated risk of bleeding.^37^^,^^39^	1a	B
	8. In the initial treatment of VTE, inferior vena cava filters can be considered when anticoagulant therapy is contraindicated. It is recommended to regularly reassess the contraindications for anticoagulant therapy, and anticoagulation should be resumed when it is deemed safe.^45^	1a	A
	9. Outpatient cancer patients who are undergoing chemotherapy and possess a Khorana score of 2 or higher, indicating a moderate to high risk of VTE, should be considered for preventive anticoagulation therapy.^33^^,^^35^, ^36^, ^37^^,^^43^^,^^48^	1b	A
	10. For outpatient lung cancer patients without a significant risk of bleeding or drug interactions, it is recommended to use apixaban, rivaroxaban, or LMWH.^39^^,^^40^^,^^43^	1b	A
	11. For hospitalized patients diagnosed with active malignant tumors or those exhibiting reduced mobility, it is advisable to implement pharmacological prophylaxis for thrombosis, provided there are no contraindications such as bleeding.^33^^,^^36^	2b	A
	12. For ambulatory patients with cancer at low risk for thrombosis receiving systemic therapy, we recommend no thromboprophylaxis over parenteral thromboprophylaxis.^40^	1a	A
	13. In the case of hospitalized cancer patients undergoing chemotherapy, the use of LMWH or unfractionated heparin is recommended for thrombosis prevention; however, DOACs are not recommended in this context.^43^^,^^41^^,^^47^	2b	A
	14. For cancer patients undergoing surgery who have no VTE, pharmacological rather than mechanical thromboprophylaxis should be adopted for those with a low bleeding risk; mechanical rather than pharmacological thromboprophylaxis should be used for those at a high bleeding risk; and for those who are undergoing surgery with a high risk of thrombosis and have a non-high bleeding risk, a combination of mechanical and pharmacological antithrombotic prophylaxis should be applied.^43^^,^^36^^,^^40^	1b	A
**Anticoagulant selection**	15. The selection of pharmacological agents is contingent upon a variety of factors. It is advisable to integrate patient preferences and values with considerations of drug feasibility, cost, risks, and benefits. Following a process of shared decision-making with the patient, an individualized treatment plan should be established.^32^^,^^35^^,^^38^^,^^40^^,^^42^^,^^45^	5a	A
	16. Before prescribing medication, it is essential to comprehensively assess patient characteristics (age, weight, hepatic/renal function, bleeding history, comorbidities, and concomitant medications), and test laboratory indices including complete blood count, PT, APTT, serum creatinine, transaminases, and bilirubin levels, to determine whether concomitant medications affect the bioavailability of the target drug.^32^^,^^39^	2a	A
	17. The primary anticoagulant agents recommended for the management of cancer-associated thrombosis are direct factor Xa inhibitors, specifically apixaban, rivaroxaban, and edoxaban. In cases where these agents are not suitable, second-line treatment options include the initiation of unfractionated heparin, followed by the administration of warfarin and fondaparinux.^32^^,^^46^	1a	B
	18. For hemodynamically stable cancer patients with VTE and adequate renal function (creatinine clearance ≥ 30 mL/min), DOACs are preferred over low-molecular-weight heparin or traditional regimens. In cases of severe renal failure (creatinine clearance< 30 mL/min), unfractionated heparin followed by vitamin K antagonists, or low-molecular-weight heparin with anti-xa monitoring, may be used.^32^^,^^36^, ^37^, ^38^, ^39^^,^^41^^,^^42^	1a	A
	19. For patients with active cancer, the net clinical benefit of DOACs is similar to or more favorable than LMWH.^47^^,^^49^	1a	A
	20. For cancer patients presenting with acute VTE and a high risk of bleeding, the administration of LMWH is recommended for treatment.^35^^,^^46^	1b	B
**Treatment duration**	21. Cancer patients diagnosed with VTE should be administered anticoagulant therapy for a duration exceeding 3–6 months. Additionally, patients who present with concurrent pulmonary embolism should receive anticoagulation treatment for a period extending beyond 6–12 months.^35^^,^^38^^,^^39^^,^^44^	2c	B
	22. For patients diagnosed with active cancer or those exhibiting persistent risk factors, the consideration of indefinite anticoagulation therapy is warranted.^32^^,^^35^	1b	A
	23. For patients with cancer undergoing perioperative care, pharmacological prophylaxis s should be started 2–12 h preoperatively and continued for at least 7–10 days.^32^	1a	A
	24. In cases where high-risk factors are identified, such as immobilization, obesity, or a prior history of VTE, it is advisable to extend the administration of LMWH for an additional 4 weeks following surgery.^36^^,^^37^^,^^39^^,^^44^	1a	A
**Recurrence prevention**	25. It is recommended to utilize DOACs for the prevention of VTE recurrence in patients with cancer.^32^^,^^40^	1a	B
	26. For patients with cancer and recurrent VTE despite anticoagulation treatment, the ASH guideline panel suggests not using an IVC filter over using a filter.^40^	1b	B
	27. In the event of VTE recurrence, three options can be considered: (1) increase LMWH by 20–25% or switch to DOACs; (2) for DOACs, switch to LMWH; and (3) for vitamin K antagonists, switch to LMWH or DOACs.^32^^,^^37^^,^^39^	1a	A
**Safety monitoring**	28. In cases where there are indications for anticoagulant prophylaxis of VTE, it is advisable to administer this therapy when the platelet count is equal to or greater than 50,000/μL. For platelet counts ranging from 25,000 to 50,000/μL, the decision should be made on an individual basis. Generally, anticoagulant prophylaxis should not be utilized when the platelet count is less than 25,000/μL.^32^^,^^33^^,^^39^^,^^43^	1b	A
	29. When bleeding occurs in patients during anticoagulant therapy, the initial step is to accurately determine the timing of the last administration of anticoagulant medications. Subsequent management should then be meticulously tailored according to the severity of the bleeding episode.^32^^,^^35^	1a	B
	30. For mild bleeding cases, it may be appropriate to defer or discontinue anticoagulant therapy. In the event of non-fatal major bleeding, immediate discontinuation of anticoagulants is required, accompanied by the implementation of comprehensive supportive measures, which may include mechanical compression, endoscopic or surgical hemostasis, fluid resuscitation, blood transfusion, and other interventions, customized to the patient's specific condition. The use of specific antagonists should also be carefully considered. In situations of life-threatening bleeding, prompt cessation of anticoagulant therapy is imperative, followed by the urgent administration of antagonists for targeted symptomatic treatment.^35^^,^^41^^,^^44^	1a	B
	31. During anticoagulant therapy, it is essential to conduct regular assessments of renal function and complete blood count to evaluate the patient's coagulation status and, when feasible, the plasma concentration of the anticoagulant medication.^32^ renal function should be monitored at a minimum of once annually; however, for specific high-risk populations, including the elderly, individuals with pre-existing renal impairment, or those receiving concurrent medications or presenting with conditions that may influence renal function, more frequent monitoring is advised.^37^^,^^48^	1b	B
**Health education**	32. Health care professionals ought to provide education to patients regarding VTE, particularly in circumstances where the risk is heightened, such as during major surgical procedures, hospitalization, and systemic anti-cancer therapies.^36^^,^^38^	1b	A
	33. Prior to the initiation of anticoagulant therapy, it is imperative that patients are adequately informed regarding the indications for treatment, the dosing regimen, and the associated risks of non-compliance. This comprehensive understanding is essential for patients to grasp both the indications and potential side effects of anticoagulants. The utilization of brochures and other educational materials is recommended to facilitate this process.^45^	1b	A
**Follow-up**	34. All cancer patients undergoing anticoagulant therapy should be monitored closely, with follow-up assessments addressing potential complications that may arise during treatment. These complications could influence the decision to continue or discontinue anticoagulation therapy and may include, but are not limited to, VTE recurrence, bleeding events, medical procedures, thrombocytopenia, the introduction of new medications, conditions impacting oral intake or drug absorption, local or metastatic progression of cancer, and the achievement of remission.^32^^,^^43^^,^^45^	1b	B

VTE, venous thromboembolism; PT, prothrombin time; APTT, activated partial thromboplastin time; LMWH, low molecular weight heparin; DOAC, direct oral anticoagulant; IVC, inferior vena cava; BNP, brain natriuretic peptide; NT-proBNP, N-terminal pro-brain natriuretic peptide; CT, computed tomography; COVID-19, coronavirus disease 2019.

## Discussion

### Main findings

Cancer-associated VTE represents a major cause of morbidity and mortality among cancer patients, and anticoagulant therapy is critical for improving patient survival.^1^^,^^2^^,^^9^ Given the poor prognosis and high medical costs of cancer patients with thrombosis, it is particularly important to determine the optimal anticoagulation treatment plan for cancer patients. Based on a comprehensive literature review and evidence assessment, this study proposes a multidimensional anticoagulant treatment protocol founded on 34 items of best available evidence. The results show that the selection and administration of anticoagulants must be individualized, as the risk of VTE varies substantially across different cancer types, cancer stages, and antineoplastic treatment modalities. Clinicians must consider multiple factors when selecting an anticoagulation regimen, including cancer type and stage, individual patient characteristics and preferences, and treatment-related factors. Shared decision-making with patients regarding anticoagulant treatment regimens can help improve treatment adherence.

This study synthesizes evidence related to VTE anticoagulation management in cancer patients across eight key domains: risk assessment, timing of anticoagulation, anticoagulant selection, treatment duration, recurrence prevention, safety monitoring, health education, and follow-up. These findings provide a basis for clinicians and nurses to standardize the management of cancer-associated thrombosis.

#### Risk assessment

Early screening and assessing high-risk factors are essential for personalized prevention, reducing recurrence and mortality, and enhancing patient quality of life.^51^ Evidence 1–5 emphasizes the risk factors, assessment contents and assessment tools that lead to hypercoagulable state in cancer patients. A case–control study conducted in the Netherlands demonstrated that the risk of VTE is the highest among patients with hematological malignancies, lung cancer, gastrointestinal cancer, brain tumors, and those with distant metastases.^52^ With the advancement of treatment regimens, multiple studies have shown a significant increase in the incidence of thromboembolic events in cancer patients treated with immune checkpoint inhibitors.^53^^,^^54^ However, these observational data derived from non-trial cohorts cannot directly infer a causal relationship between these drugs and VTE. The associated risk may be attributed to other underlying thrombotic risk factors in the patients. Given the complexity of VTE pathophysiology, currently no single risk factor or biomarker can be used alone to identify patients at high risk of VTE. The risk assessment tool for VTE needs to integrate the patient's clinical symptoms, high-risk factors, and laboratory parameters. Currently, the optimal timing for biomarker testing remains unclear, which limits its role in the continuous assessment of the disease. Imaging techniques such as Doppler ultrasound and computed tomography venography are useful for diagnosing VTE and screening high-risk patients, but they are less effective for risk assessment.^55^ For patients with solid tumors, the Khorana score is currently recommended to predict the probability of thrombosis. Nevertheless, its predictive value is controversial, particularly as it exhibits low predictive efficacy for certain specific types of cancer (e.g., lung cancer).^56^ Qin et al.^57^ developed a VTE risk assessment tool specifically for Chinese cancer patients using the Delphi-AHP method. This tool encompasses 39 different factors and has high credibility and consistency. However, further research is still needed before applying this tool in practice. However, further research is required before this tool can be implemented in clinical practice.

#### Timing of anticoagulation

For cancer patients, the addition of anticoagulant drugs to prevent VTE may increase the risk of bleeding. Therefore, it is crucial to choose the appropriate timing and the appropriate dosage of anticoagulant drugs. Evidence 6–14 summarizes the timing of anticoagulation and prevention strategies for VTE in cancer patients across different scenarios, including outpatient cancer patients, hospitalized cancer patients, and cancer patients undergoing surgery. The Chinese Guidelines for the Prevention and Treatment of Cancer-Associated Venous Thromboembolism recommends^35^ that for all hospitalized patients diagnosed with active cancer or clinically suspected of having cancer, prophylactic anticoagulation therapy may be administered after excluding peripheral arterial disease, congestive heart failure, acute superficial phlebitis, or DVT. Close monitoring of the patient's bleeding risk is required during the administration period. However, this recommendation is categorized as a Grade II recommendation with Level B evidence. In addition, for outpatient cancer patients who have initiated chemotherapy, a Khorana score of ≥ 2, no drug–drug interactions, and no high bleeding risk, rivaroxaban is recommended for primary thrombosis prevention.^35^^,^^37^^,^^48^ A study showed that for the long-term treatment of VTE in cancer patients, LMWH may significantly reduce the risk of VTE compared with vitamin K antagonists. In contrast, DOACs may reduce the risk of VTE but may increase the risk of bleeding when compared with LMWHs. For patients with cancer and VTE, when deciding to initiate long-term treatment with LMWH versus oral anticoagulants, the benefits and risks should be weighed.^9^ Additionally, the patient's values and preferences regarding outcomes, as well as alternative management strategies, should be taken into consideration.

#### Anticoagulant selection

The selection of appropriate anticoagulant medications is of paramount importance for balancing efficacy and safety. Proper drug choice can lower the risk of thrombus and recurrence while minimizing side effects like bleeding, allowing for personalized prevention plans that enhance patient compliance and quality of life. Evidence 15–20 emphasizes that when choosing a medication, factors such as the patient's preferences, liver and kidney functions, and bleeding risks should be taken into consideration. It also suggests that patients with normal kidney function should use DOACs, while those with a high bleeding risk should use LMWH. However, for patients with specific comorbidities, the selection of drugs failed to adequately consider the impact of different cancer types on drug metabolism and sensitivity. The risk assessment for combining anticoagulants and targeted drugs is lacking, often relying on empirical adjustments, and there are no clear recommendations for timing the switch between long-acting and short-acting anticoagulants during various treatment stages. Future research should focus on large-scale studies to assess the efficacy and safety of various anticoagulants for patients with specific comorbidities, addressing current evidence gaps. While LMWH is the standard for treating cancer-related VTE, its daily injections and high costs may lead patients to prefer oral anticoagulants.^58^ However, DOACs require careful consideration of interactions with anticancer and supportive care drugs.^46^ Additionally, patients' willingness to undergo anticoagulant therapy often depends on their expected survival, with those in hospice care likely opting to avoid or stop treatment. Therefore, it is advised to collaboratively create treatment plans with patients, weighing the pros and cons of anticoagulants alongside their values and preferences.^59^^,^^60^ By incorporating AI to integrate clinical data, treatment options, and drug metabolism, we can achieve precise, personalized anticoagulant selection and offer practical clinical guidance.

#### Treatment duration and recurrence prevention

The duration of anticoagulant therapy and the prevention of recurrence are critical components in the long-term management of cancer-associated VTE. However, due to the heterogeneity of these studies, there remains ongoing debate regarding the optimal duration of such therapy for cancer patients.^61^^,^^62^ Most clinical guidelines advocate for a minimum duration of anticoagulant therapy of 3–6 months for patients with active cancer, with recommendations to consider extending the treatment period. Notably, Francis's study highlights that the risk of thrombotic complications persists significantly beyond 6 months, and extending anticoagulant therapy may effectively reduce the risk of VTE recurrence.^63^ The decision to either discontinue or prolong treatment following the initial 3–6 months should be predicated on a careful evaluation of the balance between the risk of VTE recurrence and the potential for bleeding complications, alongside considerations of patient preferences, life expectancy, and treatment costs. Evidence suggests that, in the context of long-term anticoagulant therapy, direct oral anticoagulants may be associated with a reduction in thrombotic recurrence compared to LMWH; however, they may also elevate the risk of major bleeding, particularly in patients diagnosed with gastrointestinal malignancies.^64^ Consequently, it is advisable to implement individualized anticoagulant therapy tailored to the specific cancer type of each patient. This approach should include regular assessments of the risk-benefit ratio, continuous monitoring of relevant indicators such as liver and kidney function, judicious adjustments of drug dosages, and efforts to minimize the bleeding risks associated with anticoagulant use.

#### Safety monitoring

Anticoagulant monitoring is a core guarantee for the safe management of cancer-related VTE. It provides a basis for adjusting drug doses or discontinuing medication, minimizes the dual risks of bleeding and thrombus recurrence, and directly affects treatment safety and patient compliance. Studies have found that despite adequate treatment, cancer patients have a higher risk of recurrent thromboembolism and bleeding-related events during anticoagulant therapy compared with non-cancer patients.^62^ However, anticoagulants themselves do not cause bleeding; they only interfere with the normal hemostatic process. The main reasons for this are that cancer and its treatment can reduce platelets, increase inflammatory cytokines, and damage vascular integrity at the primary tumor site or metastases, thereby increasing the risk of bleeding. The evidence from this study clarifies the key indicators for drug monitoring, such as initiating anticoagulation when the platelet count is ​≥ ​ 50,000/μL, regularly assessing renal function, and principles for managing bleeding. Nevertheless, there is a lack of unified standards for specific monitoring indicators of different anticoagulants such as DOACs and LMWH; the monitoring frequency for special populations such as patients with hepatic or renal insufficiency and elderly patients has not been refined, which may lead to insufficient or excessive monitoring; the correlation between dynamic assessment of bleeding risk and monitoring indicators is not clear, and clinical decisions still rely on experience; and there is a lack of guidance on emergency monitoring procedures for sudden coagulation abnormalities during anticoagulation. Future research can focus on special populations to clarify individualized monitoring frequencies and indicator combinations; and build real-time monitoring systems with the help of wearable devices or point-of-care testing technologies, so as to provide more operable safety management strategies for clinical practice.

#### Health education and follow-up

Effective health education boosts patient awareness of thrombus risks and anticoagulant therapy, improving treatment compliance. Standardized follow-up quickly identifies complications, aiding in treatment adjustments and ensuring safe anticoagulant prevention. While the study outlines key health education content and necessary follow-up attention, it lacks refined communication strategies for patients with varying educational levels, potentially affecting information transmission effectiveness. A study by Benelhaj et al.^65^ found that most patients lack awareness of the risk of cancer-related thrombosis, and even mistakenly attribute VTE symptoms to cancer or other comorbidities, resulting in delayed consultation and diagnosis and increased patient burden. In addition, the survey also found that health care providers have a low awareness of the signs and symptoms of VTE and often cannot effectively educate patients about VTE. Given that cancer patients have different needs for VTE at different stages of the disease, it is recommended that before the diagnosis of cancer-related VTE, medical staff should strengthen the education on knowledge related to the signs and symptoms of VTE to ensure that patients understand the importance of VTE prevention.^66^ At the time of initial diagnosis and treatment of cancer-related VTE, patients should be clearly informed of the treatment indications, anticoagulant regimens, drug risks, and other contents. Adequate education helps to improve patients' self-management ability and reduce their psychological distress. DOACs offer convenience as they don't need lab monitoring, but this can lead to lower patient adherence. Regular follow-ups enhance the safety and adherence of anticoagulant therapy. The best follow-up schedule for long-term anticoagulant users is uncertain, though many doctors and guidelines suggest regular evaluations, such as monthly or quarterly, to weigh the risks and benefits. Thus, health care providers should closely monitor cancer patients with VTE, quickly assess for recurrence, contraindications, bleeding risks, and medication changes, and use blood tests and ultrasounds to identify new thrombus risks, enabling the creation of effective anticoagulant plans and improving care quality.

### Implications for nursing practice and research

This study summarizes the best evidence for anticoagulation therapy for cancer-related VTE, covering aspects such as risk factor assessment, selection of anticoagulant drugs, timing of use, treatment duration, drug monitoring, prevention of recurrence, health education, and follow-up. It provides scientific basis for clinical anticoagulation treatment decisions. In clinical practice, nurses should play a leading role: through systematic training, nurses should be aware of the limitations of the Khorana score and master the multidisciplinary dynamic assessment methods. When a patient is admitted, nurses should collect detailed medical history, pay attention to symptoms and signs, and integrate information through communication with the multidisciplinary team to lay the foundation for accurate assessment. In terms of personalized treatment support, nurses should explain the key points of anticoagulant drug use in detail, respect patient preferences, and provide psychological support to improve compliance. They need to closely monitor coagulation function, signs of bleeding, and recurrence, establish safety records, and promptly communicate and coordinate with the multidisciplinary team to adjust the treatment plan. They should carry out continuous and targeted health education, strengthen guidance during hospitalization, update patients' knowledge after discharge through lectures or online courses, and establish a dynamic follow-up mechanism to collect feedback. By formulating personalized anticoagulation plans, addressing compliance issues, regularly evaluating the quality of care, and optimizing processes, the nursing service and treatment effect can be continuously improved.

### Limitations

Although this study provides a comprehensive evidence-based framework for the management of cancer-related VTE, there are some limitations. Language restrictions may result in the exclusion of high-quality studies not published in Chinese or English. Some evidence, such as the influence of patient preferences on treatment decision-making and the best follow-up plan, lacks high-level empirical support, and relevant recommendations mostly rely on clinical experience and expert consensus, which has low credibility compared with empirical research. Risk assessment tools (such as the Khorana score) are not sensitive to specific cancers and lack more comprehensive assessment methods, which increases the risk of misdiagnosis and missed diagnosis in high-risk patients. Future research should address the following areas: 1) conduct more high-quality randomized controlled trials, focusing on exploring personalized anticoagulation regimens for patients with different cancer types and at different treatment stages, and clarifying the optimal duration of anticoagulation treatment; 2) develop and validate more precise VTE risk assessment tools to enhance the risk prediction ability for specific cancers such as lung cancer and pancreatic cancer, reducing misdiagnosis and missed diagnosis; 3) carry out multi-center and cross-regional studies to establish a clinical decision-making system that takes into account both resource accessibility and cost-effectiveness.

## Conclusions

This study systematically summarizes the best evidence for the anticoagulant management of cancer-associated VTE, providing an evidence-based reference for clinical health care providers and decision-makers. In practical application, it is necessary to consider individual patient conditions, such as cancer type, bleeding risk, and drug metabolism, evaluate the applicability of the evidence, and formulate plans that are compatible with China's medical status and patient needs. Given that most of the study literature is sourced from international databases, localized evidence is relatively insufficient. It is recommended that domestic health care providers conduct comprehensive and dynamic assessments of cancer patients, including cancer stage, VTE risk, coagulation function, and platelet count, to develop personalized anticoagulant regimens. Future research should integrate evidence-based data, clinical experience and patient preferences, carry out high-quality research, expand the existing evidence, and make the best anticoagulant therapy for patients to improve their treatment compliance.

## CRediT authorship contribution statement

**Xiang Xinyue:** Conceptualization, Methodology, Writing – Original Draft. **Yu Yudi:** Data collection, analysis and interpretation, Writing – Original draft preparation. **Zheng Tian:** Data Curation, Formal Analysis. **Fang Xiaomei:** Writing – Revised draft preparation. **Qiao Wenbo:** Project Administration, Funding Acquisition. All authors have read and approved the final manuscript.

## Ethics statement

Not required.

## Data availability statement

The data that support the findings of this study are available from the corresponding author, WQ, upon reasonable request.

## Declaration of generative AI and AI-assisted technologies in the writing process

No AI tools/services were used during the preparation of this work.

## Funding

This study was supported by the 10.13039/501100017594Medical Science and Technology Project of Zhejiang Province (Grant No. 2025KY812, Grant No. 2021KY692); Nursing Discipline Construction Research Fund (Grant No. 2025ZYHL13, Grant No. 2024ZYHL19). The funders had no role in considering the study design or in the collection, analysis, interpretation of data, writing of the report, or decision to submit the article for publication.

## Declaration of competing interest

The authors declare no conflict of interest.
