# Supplementary material for: Best evidence summary on anticoagulant management in patients with cancer-associated venous thromboembolism
Source: Asia Pac J Oncol Nurs. 2025 Sep 20;12:100789. doi: 10.1016/j.apjon.2025.100789 (PMC12547929; doi:10.1016/j.apjon.2025.100789)
Supplement: Multimedia component 1 [file mmc1.docx]

**Appendix 1**

**Search strategy**

| **Date of search** | **Search strategy** | **Database** | **Results** |
| --- | --- | --- | --- |
| 10/11/2024 | **Title/Abstract:** （“Neoplasm” OR “Tumor” OR “Cancer” OR “Malignant Neoplasm” OR“Malignancy”) AND （“Venous Thrombosis” OR “Phlebothrombosis”OR “Thrombosis, Venous” OR “Deep Vein Thrombosis” OR “Deep-Venous Thrombosis” OR “Venous Thromboses, Deep” OR “Pulmonary Embolisms”OR “Pulmonary Thromboembolisms” OR “Thromboembolism, Pulmonary”）AND （“Heparin, Low-Molecular-Weight”OR LMWH OR “Low Molecular Weight Heparin”OR“Factor Xa Inhibitors” OR “Anticoagulants, Direct-Acting Oral”OR“New oral anticoagulant”OR“Warfarin”OR“heparin”OR “unfractionated heparin”OR “Fondaparinux”OR “Dalteparin”OR“Apixaban”OR“edoxaban”OR“Vitamin K antagonist”） | Pubmed | 1458 |
| 10/11/2024 | **Title Abstract Keyword:** “Tumor OR Cancer OR “Malignant Neoplasm” OR “Malignancy” AND “Phlebothrombosis” OR “Thrombosis, Venous” OR “Deep Vein Thrombosis” OR “Deep-Venous Thrombosis” OR “Venous Thromboses, Deep” OR “Pulmonary Thromboembolisms” OR “Thromboembolism, Pulmonary” AND“Heparin, Low-Molecular-Weight” OR LMWH OR “Low Molecular Weight Heparin” OR “Factor Xa Inhibitors” OR “Direct Factor Xa Inhibitors” OR “New oral anticoagulant” OR “Warfarin” OR “unfractionated heparin” OR heparin OR Fondaparinux OR Dalteparin OR Apixaban OR edoxaban OR “Vitamin K antagonist” | Cochrane library | 45 |
| 10/11/2024 | **Abstract: (**Tumor OR Neoplasm OR Cancer OR “Malignant Neoplasm” OR “Malignancy”) AND (“Venous Thrombosis” OR “Phlebothrombosis” OR “Thrombosis, Venous” OR “Deep Vein Thrombosis” OR “Deep-Venous Thrombosis” OR “Venous Thromboses, Deep” OR “Pulmonary Embolisms” OR “Pulmonary Thromboembolisms” OR “Thromboembolism, Pulmonary”) AND (“Heparin, Low-Molecular-Weight” OR LMWH OR “Low Molecular Weight Heparin” OR “Factor Xa Inhibitors” OR “Direct Factor Xa Inhibitors” OR “New oral anticoagulant” OR “Warfarin” OR “unfractionated heparin” OR heparin OR Fondaparinux OR Dalteparin OR Apixaban OR edoxaban OR “Vitamin K antagonist”) | Web of Science | 770 |
| 10/11/2024 | **Title/Abstract:** (tumor:ab,ti OR neoplasm:ab,ti OR cancer:ab,ti OR 'malignant neoplasm':ab,ti OR 'malignancy':ab,ti) AND ('venous thrombosis':ab,ti OR 'phlebothrombosis':ab,ti OR 'thrombosis, venous':ab,ti OR 'deep vein thrombosis':ab,ti OR 'deep-venous thrombosis':ab,ti OR 'venous thromboses, deep':ab,ti OR 'pulmonary embolisms':ab,ti OR 'pulmonary thromboembolisms':ab,ti OR 'thromboembolism, pulmonary':ab,ti) AND ('heparin, low-molecular-weight':ab,ti OR lmwh:ab,ti OR 'low molecular weight heparin':ab,ti OR 'factor xa inhibitors':ab,ti OR 'direct factor xa inhibitors':ab,ti OR 'new oral anticoagulant':ab,ti) | Embase | 324 |
| 10/15/2024 | “Neoplasm” AND “Venous Thrombosis” OR “Pulmonary Embolisms” AND “Heparin, Low-Molecular-Weight” OR “Factor Xa Inhibitors” OR “Warfarin” OR “heparin” OR “Fondaparinux”OR “Dalteparin” | CNKI | 558 |
| 10/15/2024 | “Neoplasm” AND “Venous Thrombosis” OR “Pulmonary Embolisms” AND “Heparin, Low-Molecular-Weight” OR “Factor Xa Inhibitors” OR “Warfarin” OR “heparin” OR “Fondaparinux”OR “Dalteparin” | WanFang | 323 |
| 10/15/2024 | “Neoplasm” AND “Venous Thrombosis” OR “Pulmonary Embolisms” AND “Heparin, Low-Molecular-Weight” OR “Factor Xa Inhibitors” OR “Warfarin” OR “heparin” OR “Fondaparinux”OR “Dalteparin” | VIP | 157 |
| 10/15/2024 | “Neoplasm” AND “Venous Thrombosis” OR “Pulmonary Embolisms” AND “Heparin, Low-Molecular-Weight” OR “Factor Xa Inhibitors” OR “Warfarin” OR “heparin” OR “Fondaparinux”OR “Dalteparin” | Sinomed | 110 |
| 10/16/2024 | “Neoplasm” AND “Venous Thrombosis” OR “Pulmonary Embolisms” | UpToDate | 17 |
| 10/16/2024 | “Neoplasm” AND “Venous Thrombosis” OR “Pulmonary Embolisms” | Guideline | 73 |
| 10/16/2024 | “Neoplasm” AND “Venous Thrombosis” OR “Pulmonary Embolisms” | professional association websites | 68 |
|  |  | Total | 3903 |
